# Different divergence processes of isoglosses of folk nomenclature between wild trees and rice landraces imply the need for different conservation planning based on the type of plant resources

**DOI:** 10.1186/s13002-024-00675-y

**Published:** 2024-03-14

**Authors:** Yoshinori Tokuoka, Mincheol Seo, Hiroshi Hayakawa, Fukuhiro Yamasaki, Kenichiro Kimura, Kenji Takashima, Kiyokazu Hashigoe, Hiromitsu Matsui, Mitsunori Oka

**Affiliations:** 1 Faculty for Collaborative Regional Innovation, Ehime University, 3, Bunkyo, Matsuyama, Ehime 790-8577 Japan; 2 Faculty of Law and Letters, Ehime University, 3, Bunkyo, Matsuyama, Ehime 790-8577 Japan; 3 Curatorial Division, Museum of Natural and Environmental History, 5762, Oya, Suruga, Shizuoka, Shizuoka 422-8017 Japan; 4 Research Center of Genetic Resources, National Agriculture and Food Research Organization, 2-1-2 Kannondai, Tsukuba, Ibaraki 305-8602 Japan; 5 Institute for Rural Engineering, National Agriculture and Food Research Organization, 2-1-6 Kannondai, Tsukuba, Ibaraki 305-8609 Japan; 6 Sadamisaki Hanto Museum, 293 Shionashi Otsu, Ikata, Nishiuwa, Ehime 796-0506 Japan; 7 Center for Research in Science Education, Ehime University, 3, Bunkyo, Matsuyama, Ehime 790-8577 Japan; 8 Morimichi, 1-7, Teppo, Matsuyama, Ehime 790-0827 Japan; 9 Tokyo NODAI Research Institute, Tokyo University of Agriculture, 1-1-1, Sakuragaoka, Setagaya, Tokyo, 156-8502 Japan

**Keywords:** Cultural diffusion, Dialect divergence, Ethnolinguistics, Farmers’ variety, Vernacular name

## Abstract

**Background:**

The intensification of production and socio-economic changes have accelerated the loss of local traditional knowledge and plant resources. Understanding the distribution and determinants of such biocultural diversity is essential in planning efficient surveys and conservation efforts. Because the concept of biocultural diversity in socio-ecological adaptive systems comprises biological, cultural, and linguistic diversity, linguistic information should serve as a surrogate for the distribution of local biological and cultural diversity. In this study, we spatio-linguistically evaluated the names of local trees and rice landraces recorded in Ehime Prefecture, southwestern Japan.

**Methods:**

Hierarchical clustering was performed separately for the names of local trees and rice landraces. By considering innate flora differences and species having multiple local names, a novel distance index was adopted for local tree names. For the names of rice landraces, Jaccard distance was adopted. V-measure and factor detector analysis were used to evaluate the spatial association between the isogloss maps of the folk nomenclature derived from the clustering and multiple thematic maps.

**Results:**

Local tree names showed stronger spatial association with geographical factors than rice landrace names. One folk nomenclature group of trees overlapped well with the slash-and-burn cultivation area, suggesting a link between the naming of trees and the traditional production system. In contrast, rice landraces exhibited stronger associations with folklore practices. Moreover, influences of road networks and pilgrimages on rice landraces indicated the importance of human mobility and traditional rituals on rice seed transfer. High homogeneity and low completeness in the V-measure analysis indicated that the names of local trees and rice landraces were mostly homogenous within current municipalities and were shared with a couple of adjacent municipalities. The isogloss maps help to illustrate how the biological and cultural diversity of wild trees and rice landraces are distributed. They also help to identify units for inter-municipal collaboration for effective conservation of traditional knowledge related to those plant resources and traditional rice varieties themselves.

**Conclusions:**

Our spatio-linguistic evaluation indicated that complex geographical and sociological processes influence the formation of plant folk nomenclature groups and implies a promising approach using quantitative lexico-statistical analysis to help to identify areas for biocultural diversity conservation.

**Supplementary Information:**

The online version contains supplementary material available at 10.1186/s13002-024-00675-y.

## Introduction

Due to the intensification of production across plant-related sectors and socio-economic changes such as the aging and depopulation of rural societies, traditional knowledge and genetic plant resources are imperiled [1, 2]. Sophisticated systems of resource management that local communities have adopted over long periods of time can provide beneficial insights for sustainable societies, particularly as they try to better adapt to uncertain climatic and socio-economic conditions in the future [3, 4]. To effectively promote the (re)discovery and use of such systems, it is important to properly delineate the geographical boundaries of biocultural diversity. For example, in the field of dialectology, multivariate analysis based on clustering using data from previous linguistic surveys has been proposed to identify areas that need to be revisited to collect additional linguistic information [5]. Biocultural diversity is shaped by the interrelationship among biological, cultural, and linguistic diversity of local communities [6]. Therefore, plant folk nomenclature data can be seen as an important source in the evaluation of the geographical boundaries of biocultural diversity.

Ethnobotanical classification and plant folk nomenclature reflect how local communities have recognized the organisms and resources around them [7, 8]. Semantics and etymology of crops or wild plant names are utilized to infer the historical pathways of plant transfer and community contacts [9–11]. As stated by Chirkova et al. [11], field crops are more universal resources, and their names indicate widespread cognate equivalents in other regions as compared to less universal local plants, including most wild plants that lack prominent economic value. Moreover, the names for the universal crop species (e.g., the rice *Oryza sativa* subsp. *japonica*, commonly called “*Ine*”) were found to be less diverse throughout Japan [12], but more diverse landraces were shared and named by local farmers until they were displaced by modern cultivars [13, 14]. Therefore, the names of rice landraces reflect a finer scale community contact of local knowledge and seed transfer for rice production. According to a study of the Finnish language [15], multiple factors such as administrative history and environmental, geographical, and cultural differences contribute to the divergence of local dialects. However, the factors that affect the process of diverging plant folk nomenclature have not yet been quantitatively evaluated.

To better predict ethnolinguistic borders for more effective planning of the conservation of biocultural diversity, we aimed to evaluate linguistic borders and the factors that affect them for two different plant resources in Japan: local trees and rice landraces. Trees are long-lived, except for some economically valuable tree species, and most of the economically less valuable wild trees might be less intended to transfer. On the other hand, rice is an annual plant that can be stored and is often shared and transferred via seeds. It was the most important crop in the Japanese economy for millennia and is still an important part of the diet in Japan. To produce new more productive and pest resistant cultivars, selection of fine seeds during harvest and introduction of new varieties from other places were recommended in writings from the early modern era [13]. These differences in plant life form and resource characteristics between trees and rice may lead to geographically different patterns of linguistic contact, and the factors that affect the patterns may be also different. Identifying such patterns and their determinants will contribute to the conservation of indigenous knowledge related to the utilization and breeding of resource plants.

This study focuses on the plant folk nomenclature in Ehime Prefecture on the island of Shikoku, in southwestern Japan, with a particular focus on rice landraces recorded by Dainippon-beikokukai [16] and local tree names documented by Tokui [17]. The Shikoku region, which includes Ehime Prefecture, has historically been influenced by the language of the Kansai region, which includes cities like Kyoto and Osaka, because the Kansai region has been the political center of Japan since ancient times [18]. Moreover, within Shikoku, Ehime Prefecture has been linguistically influenced by neighboring prefectures via land and sea routes, leading to the sub-divergence of its local language [19]. Previous dialectological studies of Ehime Prefecture produced different thematic maps showing isoglosses of different elements of the dialect, such as accents and phonemes, as well as the dialect itself [20, 21]. Given these dialectological features, this study aimed to (1) compare the isogloss maps of wild trees and rice landraces and (2) quantitatively examine the spatial associations between the isogloss maps and thematic maps of geographical and sociological factors.

We discuss the process of formation of isoglosses with respect to local trees and rice landraces and effective planning for the conservation of traditional knowledge of those plant resources.

## Materials and methods

### Study site

**Fig. 1:**
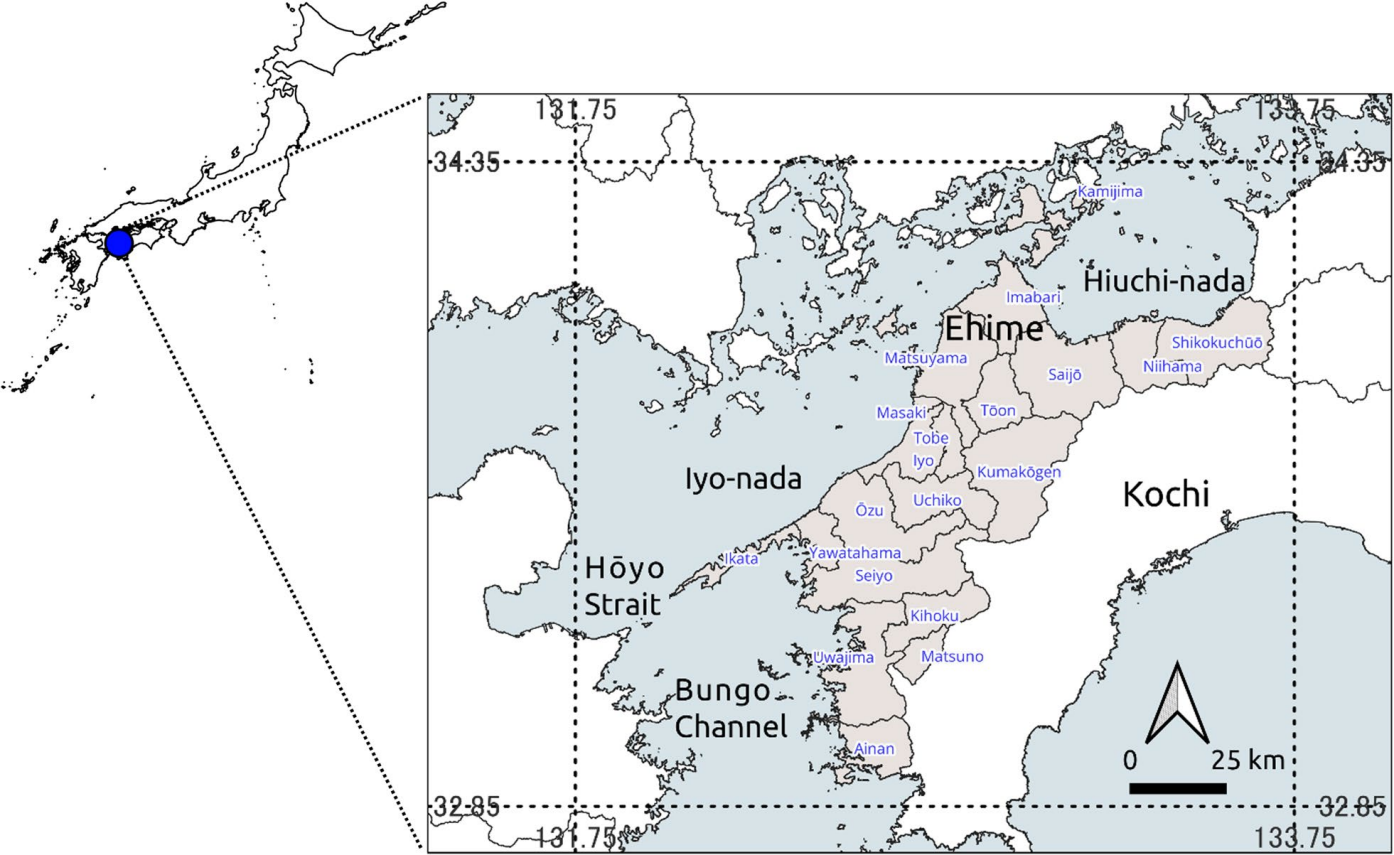
Map of the study area. The current administrative boundary data were obtained from the database of the Ministry of Land, Infrastructure, Transport and Tourism (https://nlftp.mlit.go.jp/ksj/, accessed on 12 Oct 2023)

Ehime Prefecture is in southwestern Japan and is currently divided into 20 municipalities (Fig. 1). Stretching from the coast to Mt. Ishizuchi (1,982 m above sea level), the region has significant variations in environmental conditions as shown in Additional file 1: Fig. S1A–D (altitude from [22]; precipitation, temperature, and daylight hours from [23]). The steep terrain and Hōyo Strait, the only sea route between the Uwa Sea and the Iyo-Nada Sea, have historically been geographical barriers, greatly reducing the convenience of transportation for people before motorization. Complex regional variations in folklore, which was hierarchically grouped into three levels (3, 4, and 10 groups) (Additional file 1: Fig. S1E–G from [24]), language (Additional file 1: Fig. S1H–J in [20, 21]), and agricultural practices (Additional file 1: Fig. S1K from [25]) have been illustrated as maps. The dialect map proposed by Takechi was based on the regional characteristics of grammar, vocabulary, phonemes, and accent [20]. In contrast, Sugiyama pointed out that, compared with grammar and vocabulary, the characteristics of phonemes, especially differences in accent systems, are less susceptible to influence from other dialects, and linguistic diffusion does not easily manifest [21]. He then compared the three thematic maps of dialects, phonemes, and accent, as shown in Additional file 1: Fig S1 H–J. These regional variations in various cultural characteristics may have been partly shaped by sociological factors such as restrictions on movement during the Edo period (1603–1868, Additional file 1: Fig. S1L from [26]), past road networks (Appendix Fig. S1M, https://adeac.jp/ehime-pref-lib/viewer/mp000040/MM-004/), and the changing governance structures of different eras (e.g., Appendix Fig. S1N, http://www.tt.rim.or.jp/~ishato/tiri/gun/map/1889/38ehime.htm).

### Clustering of local tree names and rice landraces

**Fig. 2:**
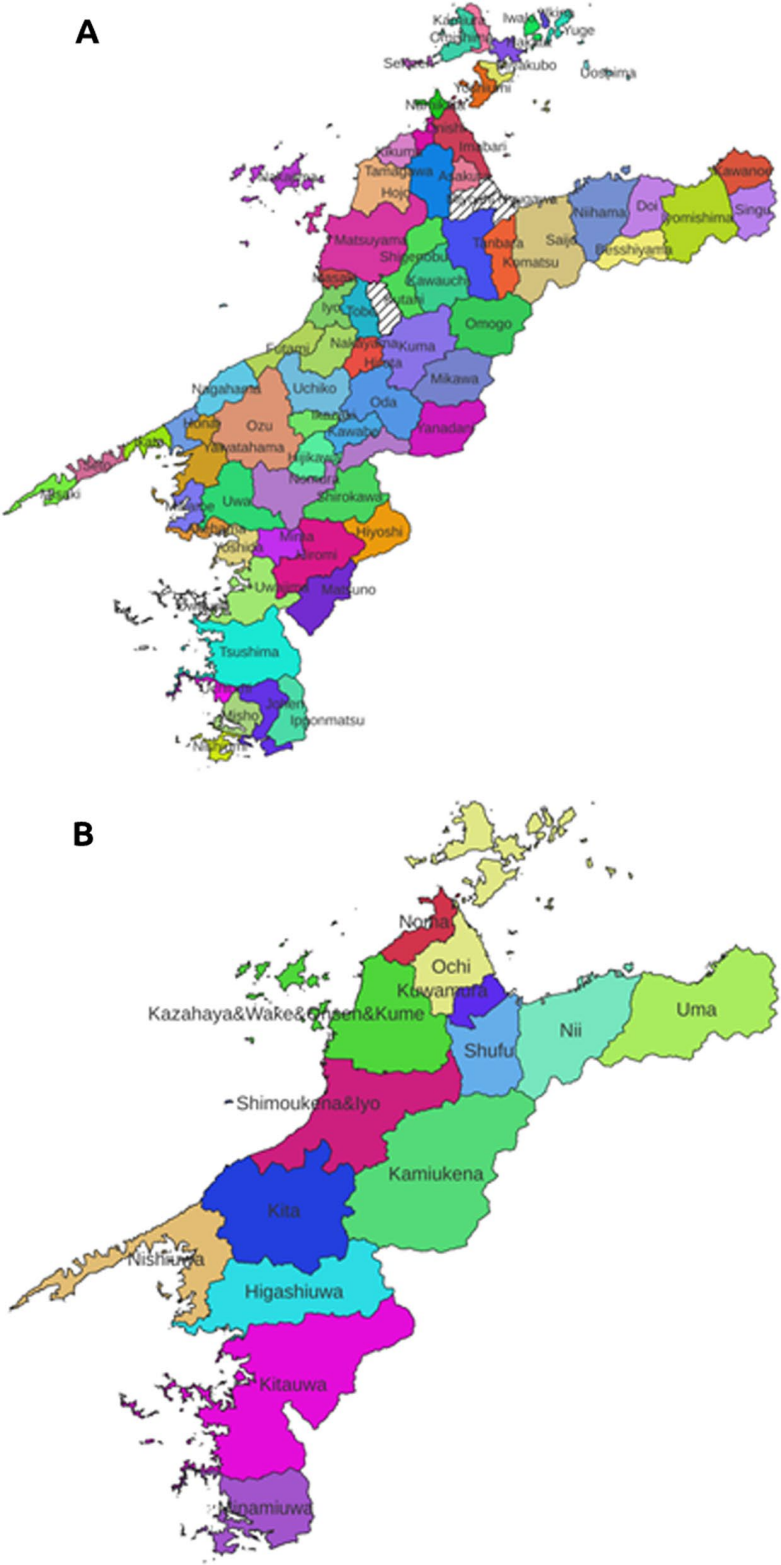
Maps showing the units of plant folk nomenclature analysis. **A** The 69 municipalities that were units of tree name analysis. **B** The 14 regions that were units of rice landrace analysis

For local tree names in Ehime Prefecture, we referred to Tokui [17]. Most of the data were collected by Mr. Tokui from the mid-1950s to 1993 by interviewing local people, and some were supplemented with other reference information. Some data were recorded in places that had been subdivided from past municipalities. During our data mining for analysis, some of these subdivided locations were integrated into the existing municipal framework of the mid-1960s. After removing three municipalities with scarce data, there was a total of 2089 local names for 310 tree species (Additional file 4: Table S1) in 69 municipalities existing in the mid-1960s. They were used for the following analysis (Fig. 2A).

Regional flora is strongly determined by ambient geographical conditions, including altitude, temperature, and precipitation. When the common target species was not selected before conducting interviews in a given survey area, as in the case of Tokui [17], innate natural differences in regional flora need to be removed to calculate the index of ethnolinguistic dissimilarity to detect the history of people’s cultural contact with wild tree resources between a certain pair of locations. In addition, multiple local plant names often exist for one species within a certain region. According to Martin [8], species with great cultural significance often have many local names, while species of less importance or with less distinctive morphological characteristics are lumped together into a single generic name. The handling of these multiple names of a species must be controlled for to equalize the weight of the species in the calculation of linguistic dissimilarity. To cope with such differences in innate regional flora and multiple names recorded for some species, the dissimilarity distance index (*D*) for the local tree name usage between a given pair of municipalities is defined as.

$$D=1 -\frac{a}{A},$$

where *a* is the number of species having at least one shared local name in the paired municipalities, and *A* is the number of species commonly included in the folk nomenclature survey of each paired municipality. This distance index was devised by referring to the Jaccard distance and the use of the percentage of shared cognates by Dyen et al. [27], which was used to estimate the time of pre-historic language splits.

We referred to Dainippon-beikokukai [16] for the local names of rice landraces. A total of 722 variety names were collected in 12 counties and two groups of counties that existed in 1885 (Fig. 2B, Additional file 5: Table S2); these names were used to calculate the Jaccard distance.

Based on these indices, hierarchical clustering by Ward linkage was applied to create isogloss maps for local tree and rice landrace names, respectively. Ward linkage was adopted because it is a merge procedure that performs well in dialectometry [28]. By interpreting the branch height and number of municipalities or counties in the clades, each of the two dendrograms was pruned hierarchically. With this procedure, it was possible to color-code the groups using similar folk nomenclature in each municipality or region, and in this paper, we recognized these color boundaries as isogloss lines.

### Indicator species analysis for rice landraces

From the clustering results, we identified characteristic rice landrace names appearing specifically in each folk nomenclature group through an indicator species analysis [29]. However, we did not conduct this analysis of the folk nomenclature of trees, which was influenced by regional differences in flora.

### V-measure and factor detector analysis

V-measure is an entropy-based measure that explicitly measures how successfully the criteria of homogeneity and completeness have been satisfied [30]. Homogeneity is evaluated by how well each cluster contains only members of a single class. Completeness is evaluated by how well all members of a given class are assigned to the same cluster. Homogeneity and completeness range between 0.0 (worst) and 1.0 (a perfect score). The V-measure is the harmonic mean of homogeneity and completeness.

Spatial stratified heterogeneity is a phenomenon in which within-strata variance is less than between-strata variance [31]. The factor detector *q*-statistic ranges between 0.0 (worst) and 1.0 (best) for the explanatory power of an explanatory variable to the response variable. This analysis was performed to explain how numerical variables (i.e., altitude, precipitation, temperature, and daylight hours) individually explain the divergence of isoglosses of folk nomenclature between local trees and rice landraces.

In our analysis, Basic Grid Squares (https://www.geospatial.jp/ckan/dataset/biodic-mesh/resource/38bd3651-120e-480f-99cf-7bb89cad7a05), each of which is approximately 1 km^2^, were overlaid on all the thematic maps of Ehime Prefecture. The hierarchical groups of our isogloss maps of folk nomenclature for local trees and rice landraces (and the features of the thematic maps of Additional file 1: Figure S1A-L) were extracted at the centroid of each grid. The extracted information at the centroids was used for V-measure and factor detector analysis. Because the thematic maps derived from various references differ in resolution and methods of original map illustration, the number of centroids extracted for certain thematic map pairs differed, but about 5,800 data points were used for each analysis.

We performed the above-mentioned analyses in R v. 4.2.3 software [32] with the ‘vegan’ [33], ‘sabre’ [30], ‘indicspecies’ [34], and ‘geodetector’ [31] packages. In the map illustrations, QGIS v. 3.28.5 software [35] was used. For the creation of isogloss maps, shapefile data from Shobunsha [36] were used.

## Results

**Fig. 3:**
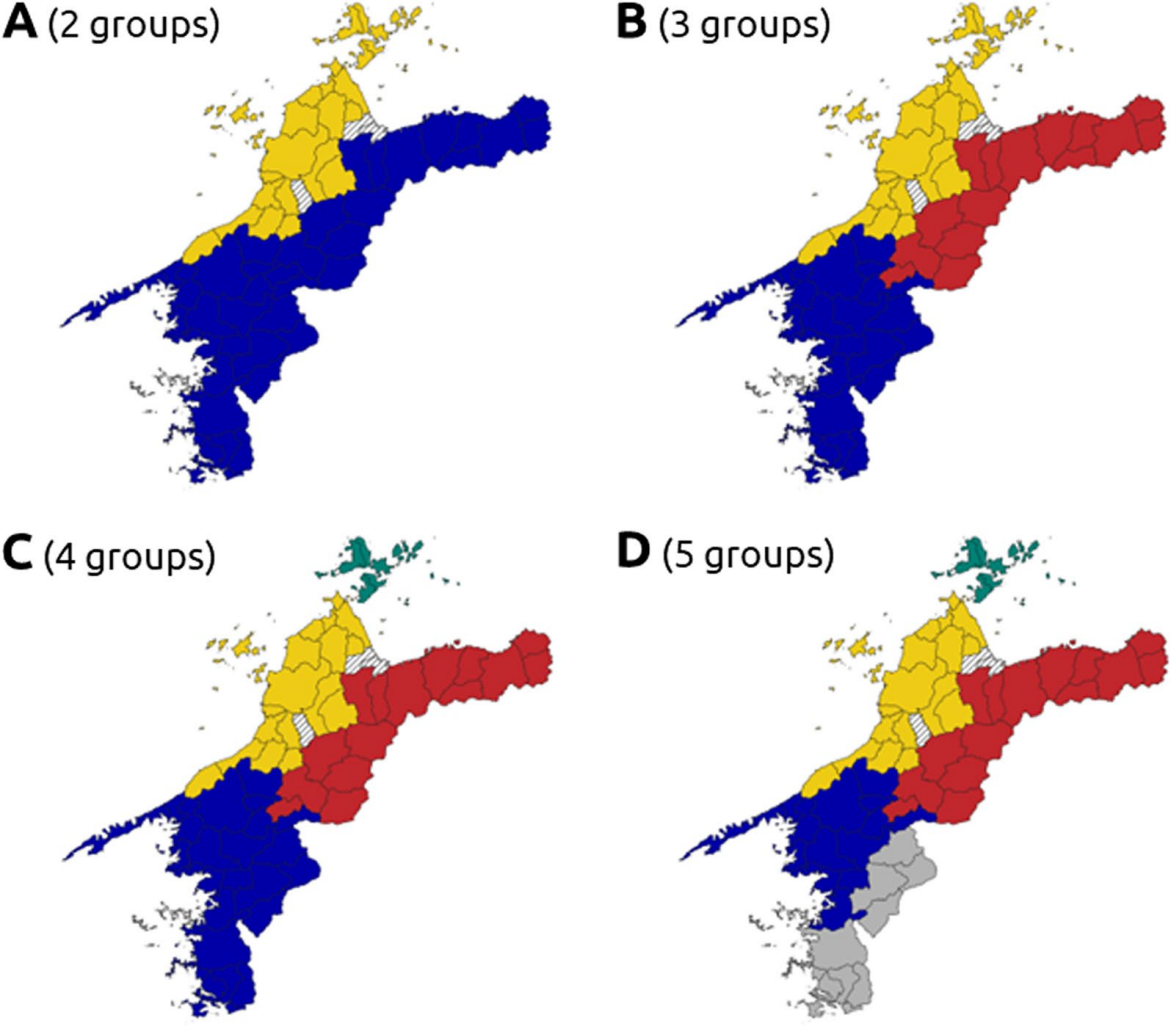
Isogloss maps obtained from hierarchical clustering using the local tree names in Ehime compiled in Tokui [17]. Group divisions for **A** 2 groups, **B** 3 groups, **C** 4 groups, and **D** 5 groups

**Fig. 4:**
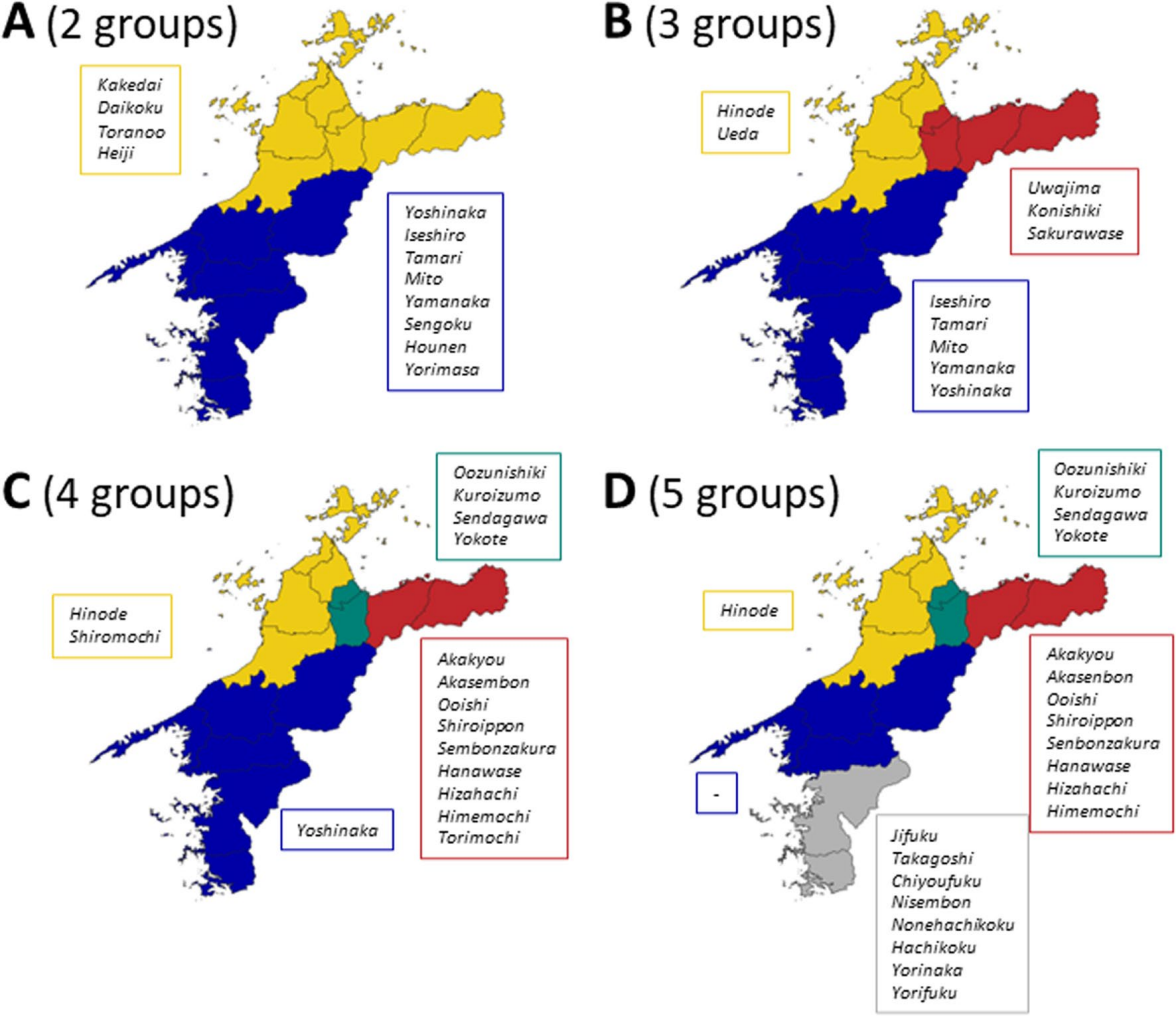
Isogloss maps obtained from hierarchical clustering using the names of rice landraces grown in 1885 in Ehime Prefecture compiled in Dainippon-beikokukai [16]. Group divisions for **A** 2 groups, **B** 3 groups, **C** 4 groups, and **D** 5 groups. Names in italics within boxes indicate the indicator variety names detected by Indicator Species Analysis, with colors corresponding to the group colors in each map

By interpreting the branch height and the number of municipalities or counties in the clades, two dendrograms were pruned at *K* = 2–5 for the names of local trees (Additional file 2: Fig. S2) and rice landraces (Additional file 3: Fig. S3). Using these groups, isogloss maps of local tree names (Fig. 3) and rice landrace names (Fig. 4) were created.

### Clustering

Although there are some limitations on direct comparison of the two maps (Figs. 3, 4) because of differences in the regional units used in the original analysis, distinct group formation patterns of folk nomenclature of local tree names and rice landraces were observed. When comparing the divergence of the maps at *K* = 2, the northeastern part of the prefecture is included in the same group as the southern and central mountainous regions in local tree names (Fig. 3A). On the other hand, in terms of rice landraces, the northeastern part of the prefecture was included in the same group as the northern islands and the lowland areas around Matsuyama, the political and economic center of the prefecture (Fig. 4A). At *K* = 3, Omogo, Kuma, Oda, and other areas in the former Kamiukena county show several differences. In local tree names, they were grouped into the eastern part of the prefecture, whereas in rice landraces, they were included in the southern part of the prefecture. At *K* = 4, the northern islands were separated from Matsuyama and its surrounding groups in local tree names. However, in rice landraces, Shufu and Kuwamura were separated from the other eastern areas of the prefecture. These two villages were located at a place where roads converge from the north, east, and west (Additional file 1: Fig. S1M). Finally, at *K* = 5, similar patterns of subdivision were observed in both local tree and rice landrace names in the southernmost part of the prefecture.

### Indicator species analysis for the folk nomenclature groups of rice landraces

Indicator species analysis identified how each hierarchical folk nomenclature groups were characterized by the specific varieties (Fig. 4). Some of the varieties included place names in and out of Ehime Prefecture such as *Uwajima*, “*Oozu*”* nishiki*, “*None*”* hachikoku* (Kochi Prefecture), *Mito* (Ibaraki Prefecture), “*Ise*”* shiro* (Mie Prefecture), and *Kuro* “*izumo*” (Shimane Prefecture).

### V-measure and factor detector analysis

No existing maps showed a very strong spatial association (i.e., > 0.7) in the V-measure evaluation results (Table 1). However, there was an intermediate to strong association (0.4–0.7) for variables other than agriculture. From the correspondence between the current municipal divisions and the isogloss maps of folk nomenclature of local trees and rice landraces, it was generally observed that homogeneity was very strong, but completeness tended to be low at *K* = 2–5. This indicates that while there is little variation in folk nomenclature within municipalities, there is a trend of specific folk nomenclature groups spanning adjacent multiple municipalities. Folklore showed a consistently strong association with rice varieties across the 3, 4, and 10 folklore divisions. For trees, however, there was only a strong association with the 10 folklore divisions and a weaker association with the 3 and 4 folklore divisions. The association between both groups of local tree names and rice landraces and accent, phoneme, standard dialect, and the Edo period political borders was weaker than that of folklore. The V-measure for agriculture, which only had two divisions, was low for both naming groups. However, the high degree of overlap between the area produced at *K* = 3–5 (the region from the east to the central mountainous part of the prefecture) and the slash-and-burn area resulted in a higher completeness value (0.45).

**Table 1:** V-measure of spatial association between social, linguistic, and economic maps and plant dialect maps. V-measure, homogeneity, and completeness range from 0 (perfectly different) to 1 (perfectly matched) between paired maps

Maps tested for association with plant folk nomenclature maps	Plant folk nomenclature maps	V-measure	Homogeneity	Completeness
Current municipality	Trees (2 groups)	0.33	0.94	0.2
	Trees (3 groups)	0.51	0.91	0.35
	Trees (4 groups)	0.53	0.88	0.38
	Trees (5 groups)	0.57	0.83	0.44
	Rice (2 groups)	0.38	0.94	0.24
	Rice (3 groups)	0.51	0.97	0.34
	Rice (4 groups)	0.52	0.93	0.36
	Rice (5 groups)	0.63	0.94	0.48
Folklore (3 groups)	Trees (2 groups)	0.25	0.31	0.21
	Trees (3 groups)	0.39	0.35	0.43
	Trees (4 groups)	0.4	0.34	0.47
	Trees (5 groups)	0.35	0.28	0.47
	Rice (2 groups)	0.69	0.78	0.62
	Rice (3 groups)	0.66	0.62	0.7
	Rice (4 groups)	0.63	0.57	0.7
	Rice (5 groups)	0.53	0.43	0.7
Folklore (4 groups)	Trees (2 groups)	0.29	0.41	0.23
	Trees (3 groups)	0.41	0.41	0.41
	Trees (4 groups)	0.42	0.39	0.45
	Trees (5 groups)	0.37	0.32	0.45
	Rice (2 groups)	0.61	0.79	0.5
	Rice (3 groups)	0.66	0.68	0.63
	Rice (4 groups)	0.65	0.65	0.66
	Rice (5 groups)	0.56	0.49	0.66
Folklore (10 groups)	Trees (2 groups)	0.36	0.8	0.23
	Trees (3 groups)	0.58	0.83	0.45
	Trees (4 groups)	0.63	0.84	0.51
	Trees (5 groups)	0.61	0.72	0.53
	Rice (2 groups)	0.44	0.86	0.29
	Rice (3 groups)	0.59	0.9	0.44
	Rice (4 groups)	0.59	0.84	0.46
	Rice (5 groups)	0.61	0.73	0.52
Accent	Trees (2 groups)	0.29	0.67	0.19
	Trees (3 groups)	0.51	0.75	0.39
	Trees (4 groups)	0.53	0.73	0.42
	Trees (5 groups)	0.54	0.65	0.46
	Rice (2 groups)	0.42	0.85	0.28
	Rice (3 groups)	0.53	0.82	0.39
	Rice (4 groups)	0.56	0.83	0.43
	Rice (5 groups)	0.64	0.79	0.54
Phoneme	Trees (2 groups)	0.21	0.36	0.15
	Trees (3 groups)	0.49	0.58	0.43
	Trees (4 groups)	0.53	0.58	0.48
	Trees (5 groups)	0.52	0.51	0.52
	Rice (2 groups)	0.45	0.7	0.33
	Rice (3 groups)	0.52	0.64	0.44
	Rice (4 groups)	0.5	0.58	0.44
	Rice (5 groups)	0.54	0.54	0.54
Grammar	Trees (2 groups)	0.18	0.34	0.12
	Trees (3 groups)	0.45	0.58	0.37
	Trees (4 groups)	0.48	0.57	0.41
	Trees (5 groups)	0.48	0.52	0.45
	Rice (2 groups)	0.39	0.67	0.27
	Rice (3 groups)	0.46	0.62	0.37
	Rice (4 groups)	0.45	0.57	0.37
	Rice (5 groups)	0.53	0.58	0.49
Agriculture (slash and burn or not)	Trees (2 groups)	0.08	0.07	0.1
	Trees (3 groups)	0.26	0.18	0.45
	Trees (4 groups)	0.24	0.16	0.45
	Trees (5 groups)	0.21	0.13	0.45
	Rice (2 groups)	0.01	0	0.01
	Rice (3 groups)	0.12	0.08	0.19
	Rice (4 groups)	0.11	0.08	0.19
	Rice (5 groups)	0.13	0.08	0.27
Tenryou and the eight domains in the last Tokugawa Shogunate	Trees (2 groups)	0.16	0.36	0.11
	Trees (3 groups)	0.42	0.59	0.33
	Trees (4 groups)	0.42	0.56	0.34
	Trees (5 groups)	0.42	0.49	0.37
	Rice (2 groups)	0.31	0.6	0.21
	Rice (3 groups)	0.39	0.57	0.3
	Rice (4 groups)	0.42	0.58	0.33
	Rice (5 groups)	0.46	0.54	0.41

**Fig. 5:**
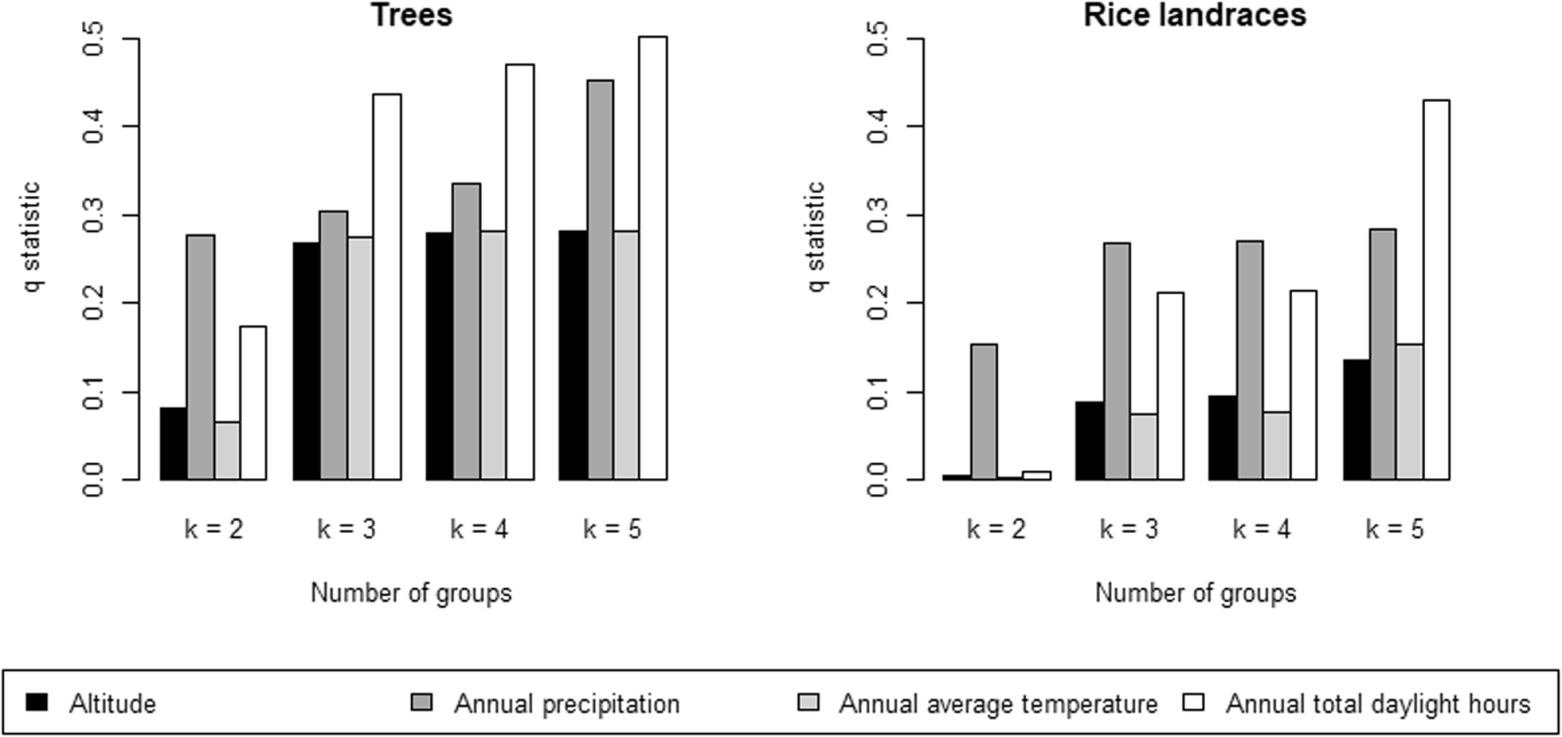
q-Values calculated in the factor detector analysis on the four geographical variables (altitude, precipitation, temperature, and daylight hours) under different numbers of groups obtained by hierarchical clustering on local tree and rice landrace names

In the factor detector analysis results, the spatial association between all folk nomenclature groups of local tree names and rice landraces and the variables of elevation, precipitation, temperature, and daylight hours never exceeded a moderate strength, reaching a maximum of less than about 0.5 (*P* < 0.05; Fig. 5). For both local tree names and rice landraces, the q-statistics for precipitation and daylight hours were relatively high among the four variables, and the spatial association with the four variables was stronger in the case of local tree names than for rice landraces.

## Discussion

### Different divergence processes of isoglosses of folk nomenclature between wild trees and rice landraces

A comparison of isoglosses of folk nomenclature between local trees and rice landraces revealed clear differences (Figs. 3, 4). Results from the V-measure and factor detector analyses suggested that the differentiation in the divergence processes of the isoglosses between the two plant resources may be attributed to different geographical and sociological variables (Table 1, Fig. 5). The divergence of local tree names showed a stronger association with geographical variables such as altitude, precipitation, temperature, and daylight hours (Table 1). Our results suggested a spatial association between the folk nomenclature group of trees and areas with slash-and-burn cultivation extending from the eastern to the central inland parts of the prefecture (Table 1, Fig. 1, Additional file 1: Fig. S1). This included the inland mountainous region in Shikoku where slash-and-burn cultivation was once widely practiced [25]. These findings may correspond to the shared knowledge of trees serving as fertilizer sources for the slash-and-burn cultivation in this area.

Unlike trees, rice is an annual plant, and rice landraces with desirable traits have been frequently introduced from region to region. Locally well-adapted varieties were selected until the mid-twentieth century [13]. During the Shikoku *Henro* Pilgrimage, in which pilgrims circumambulate the 88 temples on Shikoku Island mainly in a clockwise direction [37], rice landraces were scrutinized by pilgrim farmers in autumn, who later brought back panicles to their homelands [38]. In fact, rice landraces with a prefix or suffix related to the pilgrimage (e.g., “*Henro*” or “*Hendo*” or with the name “*Henro*” itself) were recorded in our source reference [16]. Moreover, landrace names associated with other pilgrimages to Izumo Shrine and Ise Shrine [39, 40], such as ‘*Izumo*’, ‘*Izumo-modori*’, ‘*Izumo-wase*’, ‘*Ise*’, ‘*Ise-shiro*’, and ‘*Ise-naka*’, indicated domestic seed transfers during farmers' travels. As shown in Fig. 4, indicator varieties included place names such as “*Ise*”* shiro*, *Kuro *“*izumo*”, *Uwajima*, and “*Oozu*”* nishiki*, indicating both pilgrimages and other seed transfer opportunities from outside, making the landrace composition unique in each region. Rice cultivation is closely associated with Japanese local rituals [41], which accords well with the V-measure results, which show better spatial association between the isogloss maps of rice landraces and folklore types as compared to those of local tree names.

Dialects are known to dynamically change with human mobility and be influenced by various factors, for example, infrastructure (e.g., roads) [42]. The presence of a specific group of rice landrace names at the road intersection areas of Shufu and Kuwamura, both recorded and depicted in the late nineteenth century, can be considered as an example of this dynamism.

### Implications for conservation planning

Amid Japan's economic stagnation in recent decades, there has been a push for local government mergers. Remote municipalities, especially those in mountainous areas, face challenges such as aging populations, declining population numbers, and budget constraints. Consequently, implementing concrete measures to address national environmental issues, such as the utilization of biomass energy, has proven difficult at the local municipality level [43]. Therefore, effective surveys are required to conserve agricultural biodiversity in such areas, including indigenous knowledge such as local plant names and traditional crops, while considering the resource constraints of local governments. From such a perspective, our approach is similar in concept to that proposed by Jeszenszky et al. [5], in that it utilizes clustering results of existing linguistic data. In the concept of biocultural diversity, it is thought that the diversity of life comprises of biological, cultural, and linguistic diversity in a complex socio-ecological adaptive system [6]. Under this assumption, our approach focusing on plant folk nomenclature has been shown to be effective for the rapid selection of a reduced number of survey sites to revisit for the conservation of plant resources as biological diversity assets and of local knowledge as cultural and linguistic diversity assets. According to our V-measure analysis (Table 1), high homogeneity (at *K* = 2–5) indicates that the variation in folk nomenclature of local trees and rice landraces within municipalities was not large. Conversely, the low completeness and the group formation in the maps (Figs. 3, 4) suggest that the same folk nomenclatures of local trees and rice landraces are distributed across adjacent several municipalities. Under limited human and budget resources, these results suggest how inter-municipal collaboration can be spatially designed to preserve not only linguistic diversity, but also indigenous knowledge related to those plant resources and traditional rice varieties themselves.

The results of this study provide important insights into the selection of survey areas for indigenous knowledge and traditional crops in regions without existing plant folk nomenclature data. A quantitative analysis of dialect in Finland [15] suggested a process in which multiple complex factors influence the isolation of settlements, leading to dialect divergence. The results of the V-measure and factor detector analyses in this study did not reveal any unique thematic maps strongly spatially associated with the isogloss maps of local trees and rice landraces. However, a couple of thematic maps had moderately stronger spatial associations with the isogloss maps of the two plant resources in different ways. This result may reflect a similar community isolation process governed by multiple factors [15]. If such a process prevails in other regions, it would be effective to overlay multiple available maps, such as folklore and environmental variables showing higher V-measure scores and q-statistics in this study, using the Geographic Information System and to identify possible local areas that may have a different culture of plant resource usage before conducting field surveys.

### Limitations and future challenges

In this study, hierarchical clustering by Ward linkage was applied to create isogloss maps for local tree names using the novel distance index and rice landrace names using Jaccard distance. The clustering results at *K* = 2–5 showed that all municipalities in each group were in adjacent relationships, making it easy to interpret as natural group formation. The method may have some inaccuracies as compared to dialect comparison studies that target specific balanced lexical sets. However, many folk nomenclature studies were conducted individually in specific regions according to their respective purposes, making it difficult to prepare balanced lexical sets by using these different information sources. Therefore, proposing a new dissimilarity index that enables inter-regional comparisons using such unbalanced lexical sets from various data sources is considered beneficial. Evaluation of word formative structure [44], semantics [9–11], and etymology [12] of plant names will also provide information about human contact history relative to plant resources. Moreover, reflecting cognancy and similar wording in the dissimilarity index is another lexico-statistical challenge, but the identification of cognates requires a considerable amount of time [45]. The determination of how to weigh similar vocabulary and formative structure is also a significant challenge when dealing with a vast vocabulary set. From these perspectives, the distance metric we propose in this study is versatile because it can mechanically identify groups using similar plant folk nomenclature immediately.

Much of the folk nomenclature for trees referenced in this study was recorded locally several decades ago, and rice variety names were recorded over a century ago. Consequently, much of the biocultural diversity associated with this folk nomenclature may have already been lost. Therefore, the biocultural diversity remaining today is invaluable, and efforts to collect it are urgently needed. However, there is a wealth of descriptive data of plant folk nomenclature, and representative references were compiled in [46]. Using such available information, it is desirable to test our approach in different or wider regions and verify its validity in additional studies. Fletcher et al. [47] discuss the importance of local and global economic and political efforts to expand protected areas with local linguistic and cultural connections. Our analytical approach will contribute to such efforts, particularly the use of the proposed novel dissimilarity index on plant folk nomenclature utilization for the selection of survey sites. Moreover, this study highlighted the dynamic nature of plant folk nomenclature divergence and the importance of inter-municipal collaboration in conserving indigenous knowledge and resources.

### Supplementary Information

**Additional file 1: Figure S1.** Geographical and sociological feature maps of Ehime Prefecture. A) Altitude [22]; B–D) precipitation, temperature, and daylight [23]. E–F) Folklore [25], H–I) accents and phonemes [21], J) standard dialect [20], K) slash-and-burn agriculture [26], L) the late Tokugawa Shogunate administration [24], M: roads in the Meiji period (https://adeac.jp/ehime-pref-lib/top/), N) counties in the area around 1889 (edited from https://nlftp.mlit.go.jp/ksj/jpgis/datalist/KsjTmplt-N03.html).

**Additional file 2: Figure S2** Dendrogram of 2089 local names of 310 tree species in Ehime Prefecture.

**Additional file 3: Figure S3** Dendrogram of the rice landrace name of 722 farmers’ varieties in Ehime Prefecture.

**Additional file 4: Table S1** List of 310 tree species whose local names in Tokui (1995) were used for the clustering in this study.

**Additional file 5:** **Table S2** List of the rice landrace names of 722 farmers' varieties in Ehime Prefecture in Dainippon-beikokukai (1921) that were used for the clustering in this study.

## Data Availability

The data that support the findings of this study are available from the corresponding author upon reasonable request.
