# Supplementary figures and images for: Different divergence processes of isoglosses of folk nomenclature between wild trees and rice landraces imply the need for different conservation planning based on the type of plant resources
Source: J Ethnobiol Ethnomed. 2024 Mar 14;20:35. doi: 10.1186/s13002-024-00675-y (PMC10941470; doi:10.1186/s13002-024-00675-y)

**A**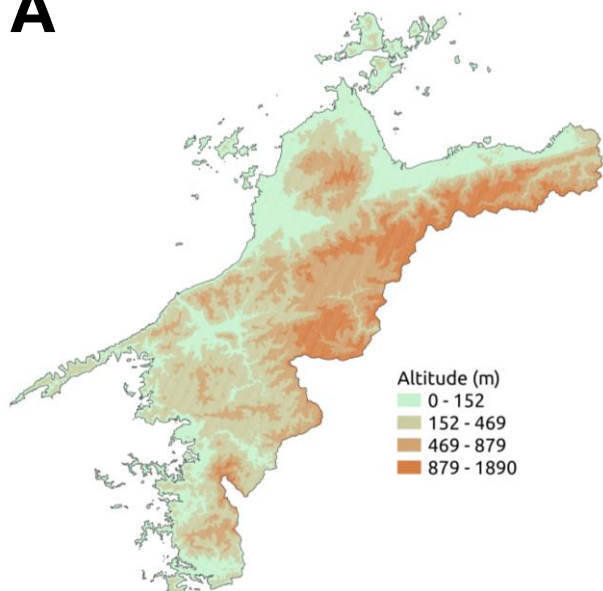**B**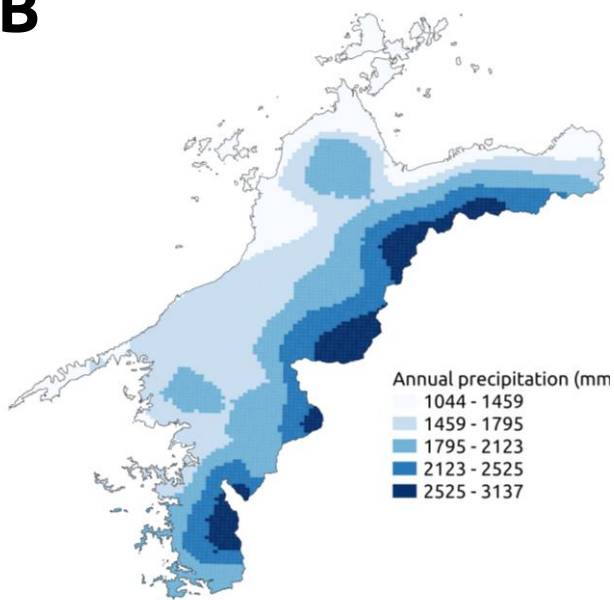**C**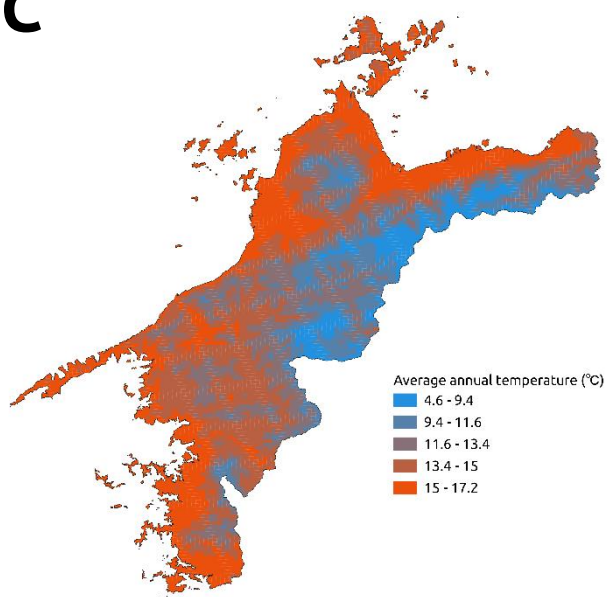**D**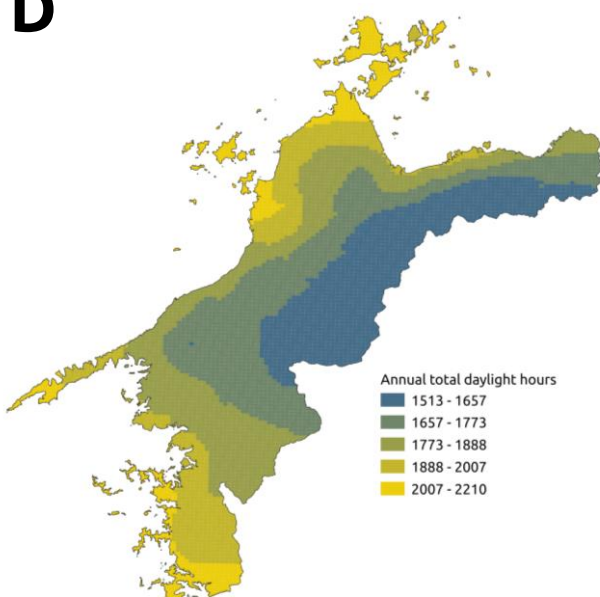**E**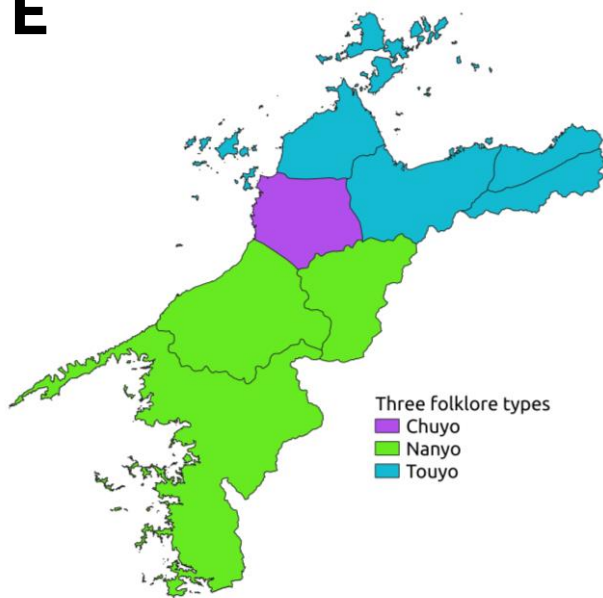**F**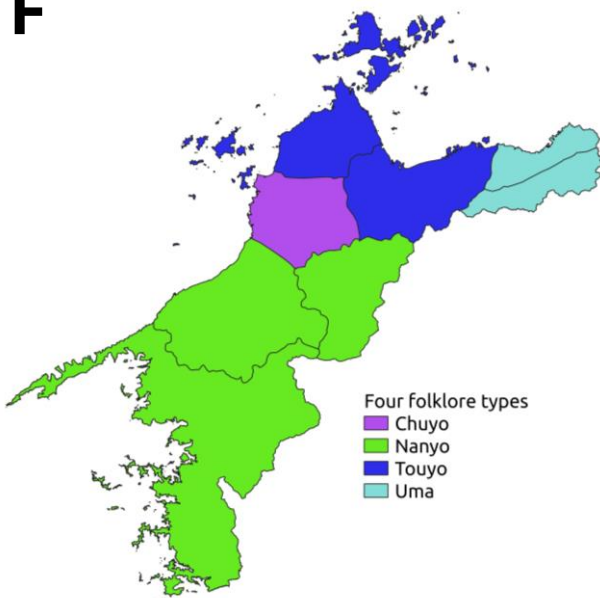

G

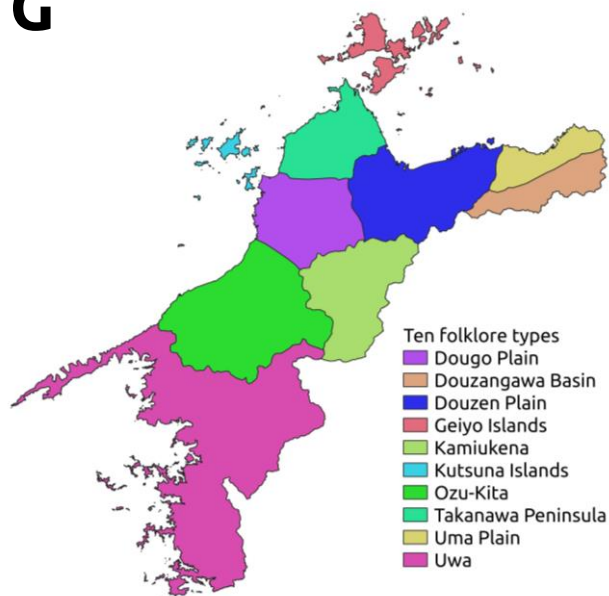

H

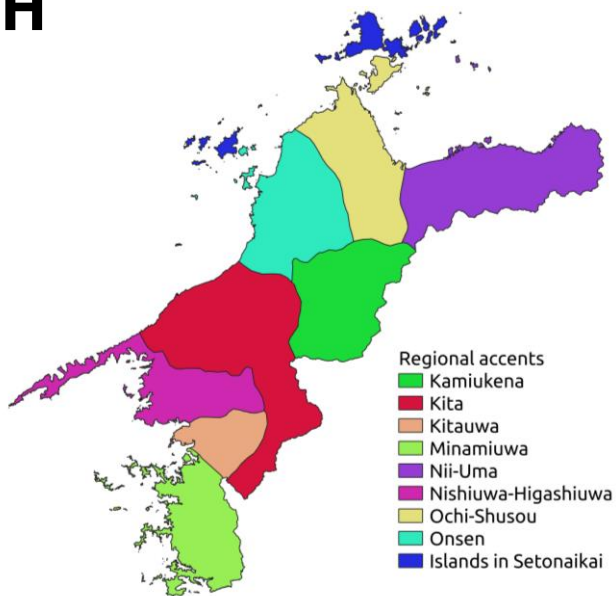

I

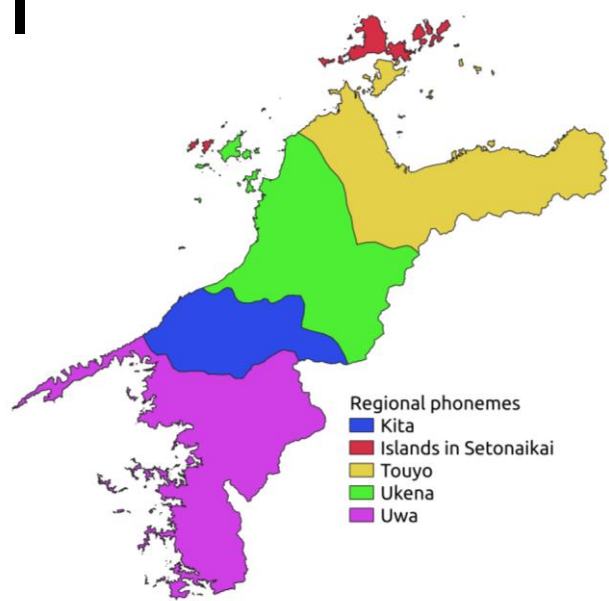

J

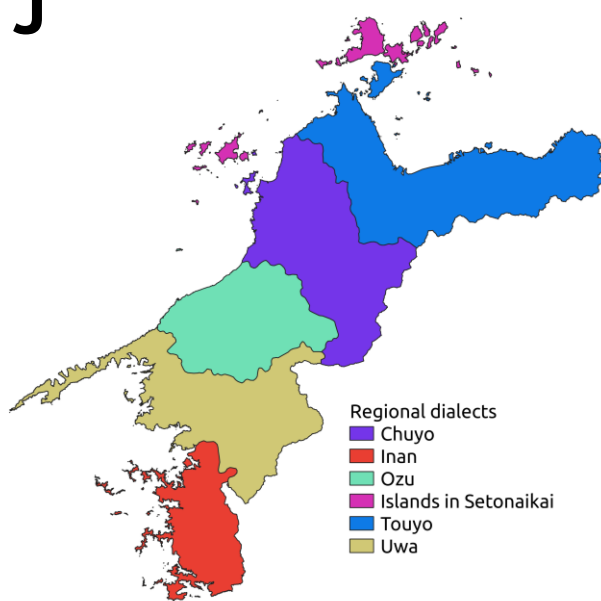

K

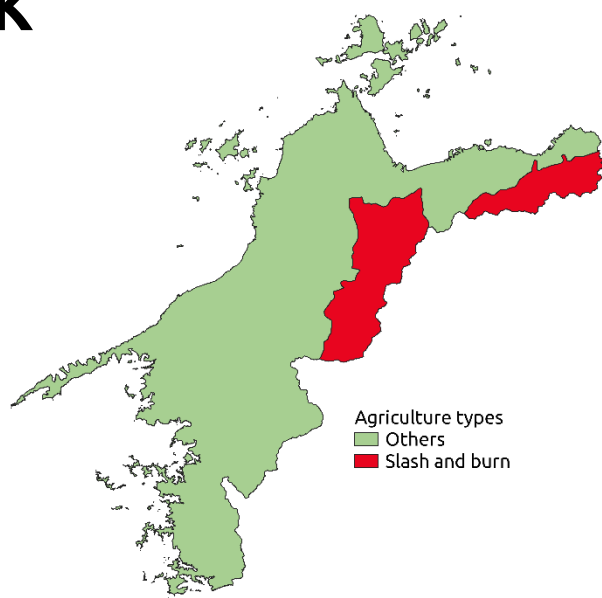

L

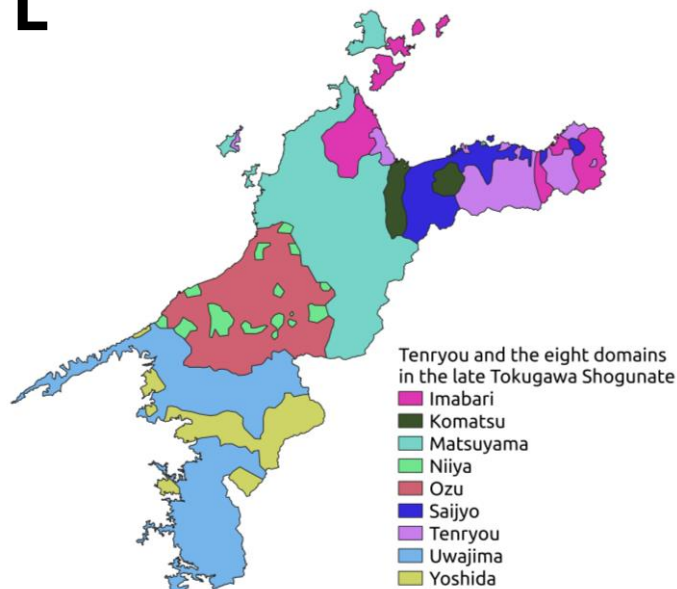

M

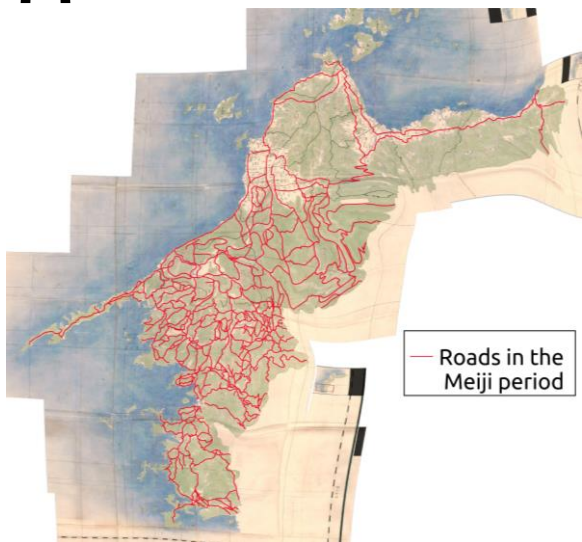

N

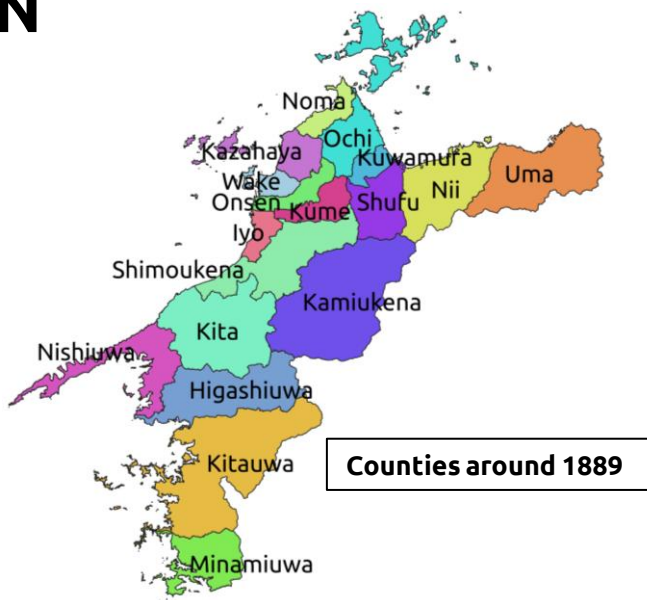

Figure S1

Supplement: Supplementary file 1 — Additional file 1: Figure S1. Geographical and sociological feature maps of Ehime Prefecture. A) Altitude [22]; B–D) precipitation, temperature, and daylight [23]. E–F) Folklore [25], H–I) accents and phonemes [21], J) standard dialect [20], K) slash-and-burn agriculture [26], L) the late Tokugawa Shogunate administration [24], M: roads in the Meiji period (https://adeac.jp/ehime-pref-lib/top/), N) counties in the area around 1889 (edited from https://nlftp.mlit.go.jp/ksj/jpgis/datalist/KsjTmplt-N03.html). [file 13002_2024_675_MOESM1_ESM.pdf]

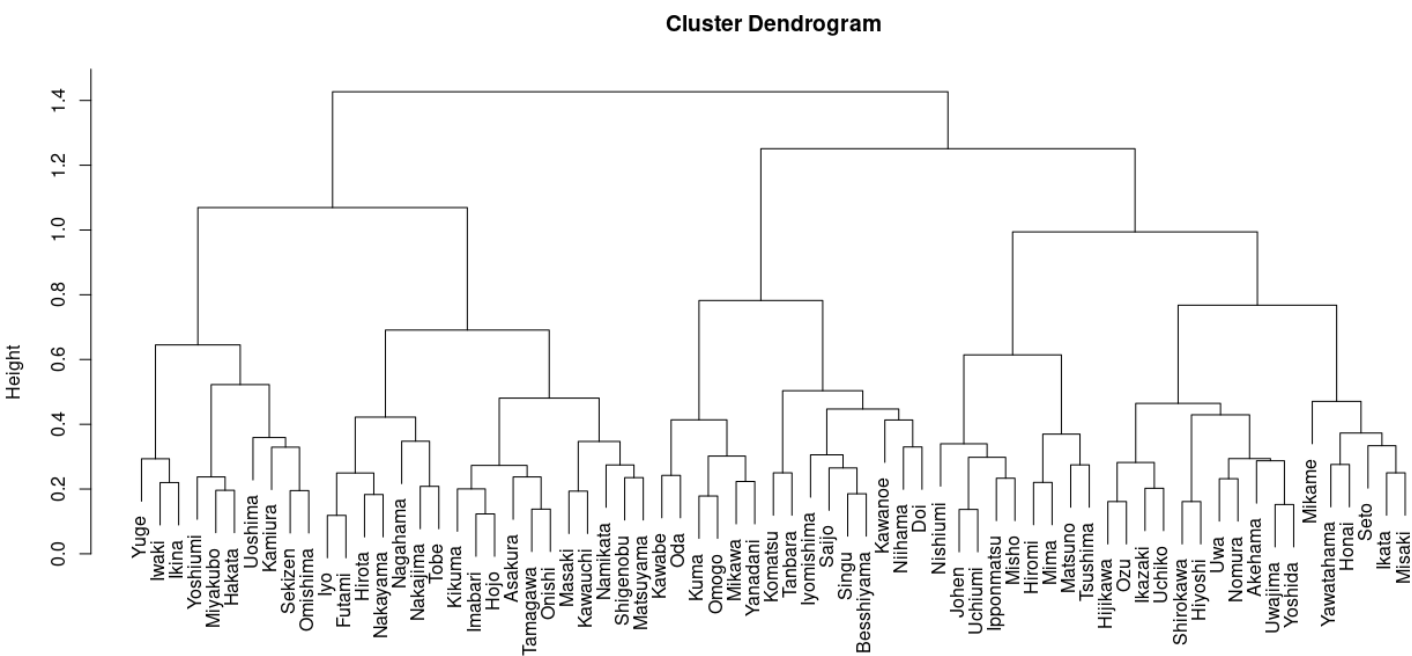

**Figure S2**

Supplement: Supplementary file 2 — Additional file 2: Figure S2 Dendrogram of 2089 local names of 310 tree species in Ehime Prefecture. [file 13002_2024_675_MOESM2_ESM.pdf]

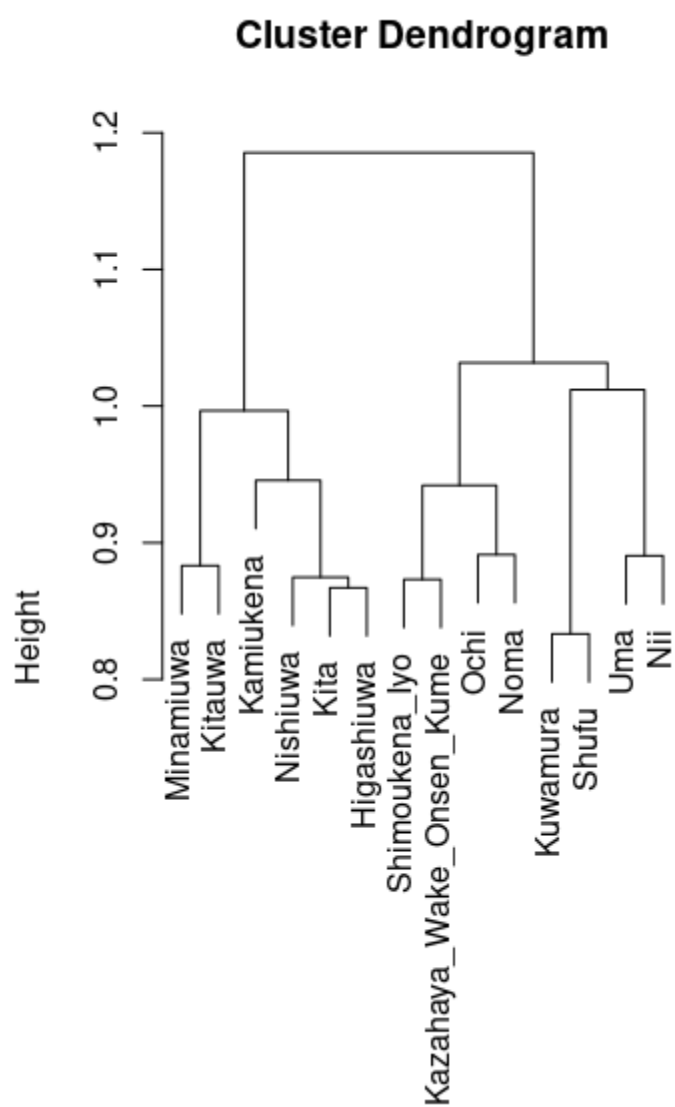

**Figure S3**

Supplement: Supplementary file 3 — Additional file 3: Figure S3 Dendrogram of the rice landrace name of 722 farmers’ varieties in Ehime Prefecture. [file 13002_2024_675_MOESM3_ESM.pdf]
